# A scoping review of the contralateral effects of unilateral peripheral stimulation on neuromuscular function

**DOI:** 10.1371/journal.pone.0263662

**Published:** 2022-02-09

**Authors:** Shi Zhou, Shuang-Shuang Zhang, Zachary J. Crowley-McHattan

**Affiliations:** 1 Discipline of Sport and Exercise Science, Faculty of Health, Southern Cross University, Lismore, New South Wales, Australia; 2 School of Sport Science, Beijing Sport University, Beijing, China; Universita degli Studi di Milano, ITALY

## Abstract

It is known that resistance exercise using one limb can affect motor function of both the exercised limb and the unexercised contralateral limb, a phenomenon termed cross-education. It has been suggested that cross-education has clinical implications, e.g. in rehabilitation for orthopaedic conditions or post-stroke paresis. Much of the research on the contralateral effect of unilateral intervention on motor output is based on voluntary exercise. This scoping review aimed to map the characteristics of current literature on the cross-education caused by three most frequently utilised peripheral neuromuscular stimulation modalities in this context: electrical stimulation, mechanical vibration and percutaneous needling, that may direct future research and translate to clinical practice. A systematic search of relevant databases (Ebsco, ProQuest, PubMed, Scopus, Web of Science) through to the end of 2020 was conducted following the PRISMA Extension for Scoping Review. Empirical studies on human participants that applied a unilateral peripheral neuromuscular stimulation and assessed neuromuscular function of the stimulated and/or the unstimulated side were selected. By reading the full text, the demographic characteristics, context, design, methods and major findings of the studies were synthesised. The results found that 83 studies were eligible for the review, with the majority (53) utilised electrical stimulation whilst those applied vibration (18) or needling (12) were emerging. Although the contralateral effects appeared to be robust, only 31 studies claimed to be in the context of cross-education, and 25 investigated on clinical patients. The underlying mechanism for the contralateral effects induced by unilateral peripheral stimulation remains unclear. The findings suggest a need to enhance the awareness of cross-education caused by peripheral stimulation, to help improve the translation of theoretical concepts to clinical practice, and aid in developing well-designed clinical trials to determine the efficacy of cross-education therapies.

## Introduction

It is known that motor practice using one limb can affect motor output in both the exercised muscle and the homologous muscle of the unexercised limb [1–3]. Several terms have been used in the literature to describe this phenomenon, such as cross education, cross training, cross transfer, or interlimb transfer, etc. However, a consensus has recently been reached among experts in this field through a Delphi survey, that “cross-education” should be used consistently in future reference to this phenomenon [3]. It should be noted that, cross-education is defined as “the increased motor output (i.e., force generation, skill) of the opposite, untrained limb following a period of unilateral exercise training” by the experts who participated in the Delphi survey [3]. This raises a question that whether the studies on the acute effect of a single bout of unilateral exercise or stimulation could be regarded as under the umbrella of cross-education. It is understandable that the adaptations to exercise training or intervention are based on the cumulative effects in response to repeated single stimulation sessions. It is important to examine and understand the acute responses and their contribution to the chronic adaptation. In this context, the definition of cross-education might be extendable to the investigations on the acute effect of a single bout unilateral exercise or stimulation. Therefore, studies that investigated either acute or chronic interventions were included in this review.

Researchers and health practitioners have had a continued interest in the cross-education because it not only raises questions about the mechanisms of neural plasticity in response to unilateral exercise, but also has clinical implications, such as in rehabilitation for paresis post stroke, or after a single limb injury or surgical operation [4–9]. In respect to physiological mechanisms, the general consensus is that cross-education is mainly manifested by adaptations in the central nervous system (CNS). The viewpoint is supported by the common finding that no significant muscle hypertrophy is associated with increased strength in the unexercised contralateral limb after a short period of unilateral training [10–14]. It has been proposed that unilateral voluntary contractions can bring about complex changes in the cortical motor pathways controlling the contralateral homologous muscle [15]. Alternatively, the neural adaptations may reside in supraspinal areas that are predominantly involved in the control of the trained limb, and these modified neural circuits may be accessed during voluntary contractions of the untrained limb [15]. It has also been hypothesised that cross-education of strength may be best applied to clinical populations with asymmetries, such as neurological damage after stroke or unilateral orthopedic injury [5].

There have been reports on the clinical efficacy of cross-education in the treatment of, and rehabilitation after, an injury, surgical operation, or stroke [4, 16, 17]. For example, a systematic review that analysed the available cross-education evidence on muscle strength in post-stroke hemiplegic patients [4] presented two eligible research articles amongst the 53 screened. Both articles reported an improved strength performance in the untrained, more affected dorsiflexor muscle after training the less affected limb. In contrast, there are reports that cross-education does not accelerate the rehabilitation of neuromuscular functions after ACL reconstruction [17, 18].

Much of the evidence mentioned above results from unilateral resistance training and/or interventions using voluntary contractions exclusively. Interestingly, some alternative training methods, such as neuromuscular electrical stimulation (NMES) or electromyostimulation (EMS) [19, 20], mechanical vibration [21–23], and acupuncture or needling [24–26], have also exhibited cross-education benefits. These interventions are loosely termed “peripheral neuromuscular stimulation” in this article, to distinguish them from voluntary resistance training and interventions that apply stimulation directly to the CNS, such as transcranial magnetic stimulation (TMS), transcranial direct current stimulation (tDCS), or similar.

It should be noted that the principle of unilateral or contralateral therapy has been applied clinically for centuries in traditional Chinese medicine. One example for the applications of this principle is acupuncture under a treatment strategy termed *juci* (contralateral meridian needling) or *miaoci* (contralateral collateral needling), that are also translated as *opposing needling* by some authors [27, 28]. Although such practices existed historically, only in the recent decades the efficacy of these *opposing needling* interventions and their potential mechanisms have been more rigorously examined in laboratory and clinical studies [29–31]. Furthermore, acupuncture and dry needling (DN) as a means of therapy has also been utilised in western countries [32, 33]. Researchers and practitioners have been critically examining the potential mechanisms and clinical efficacy of the DN, while recognising that their theoretical framework is not the same as that of acupuncture, for example, there are differences in how to determine the optimal sites and the techniques of needling between acupuncture and DN [30, 32–35].

From a health practice viewpoint, a unilateral intervention without voluntary muscle contraction, such as electrical stimulation, vibration, or needling, would have clinical implications, particularly for individuals with limited capacity in performing voluntary contractions using the affected limb. In respect to the underlying mechanisms, a compelling question is whether the contralateral effect of unilateral peripheral neuromuscular stimulation is manifested via the same or different neuromuscular mechanisms proposed for the cross-education resulting from voluntary contractions [36].

Scoping reviews are a way of knowledge synthesis that utilises a systematic approach to map evidence on a topic and identify the main concept, theories, sources, and knowledge gaps [37]. They may lead to further analysis of the evidence, such as systematic reviews and meta-analysis [37, 38]. The aim of this article was to provide a scoping review of the current literature on the contralateral effects of unilateral peripheral neuromuscular stimulations, following the recent guidelines for scoping reviews [37, 39], to summarise:

1. the demographic characteristics of the eligible literature, including the number of research articles, year of publication, country and/or laboratory where the research was conducted, context of the studies, participants, setting, and types of research design;
2. the intervention programs, including trials on acute and chronic effects, and the methods employed for peripheral stimulation and evaluation of the outcomes, including statistical analysis methods; and
3. the research aims, major findings, and limitations for the studies that claimed to be in the context of cross-education.

After a preliminary search of the literature, we found that the major types of peripheral neuromuscular stimulation being electrical stimulation, vibration, and acupuncture or needling. Therefore, this review focused mainly on these three types of stimulation modalities. The terms of electrical stimulation, vibration, and acupuncture or needling, as used in this article, are defined below.

In this review, electrical stimulation (ES) refers to the practice or methods that apply electrical impulses via surface electrodes over a peripheral nerve or a skeletal muscle [40], or through intramuscular electrodes such as in electroacupuncture [41], to evoke sensory inputs and/or motor activities, aiming to examine or improve neuromuscular function. Transcutaneous electrical stimulation has been referred to as EMS [12], NMES [40], or transcutaneous electrical nerve stimulation (TENS) [20], depending on the methodology and context. The intensity, pulse width and shape, frequency, and other stimulation parameters are controlled via an electrical stimulator [42].

Vibration (VB) refers to utilization of a vibration device to deliver forced mechanical oscillation to the human body or parts of it [43]. Small vibratory units can be placed directly on a muscle or tendon, and larger units that can elicit vibration through cables, belts, or platforms, and produce vertical sinusoidal or synchronous vibration when the participant uses or stands on the device [44].

Acupuncture or needling involves the use of sharp, thin (filiform) needles that are inserted into the body at specific points (e.g., acupoints or taut band) for the treatment of health conditions [45]. Electroacupuncture refers to applying electrical stimulation through the acupuncture needles [46]. Other practices also involve the insertion of needles to treat health conditions, such as DN [32, 47]. It is beyond the scope of this review to discuss the differences in the theoretical frameworks of the acupuncture and DN [33, 47]. From a practical viewpoint, ‘needling’ (ND) is used in this article for the practices that involve the insertion of needles percutaneously into tissues for the purpose of research, such as to examine the ergogenic effects of needling on athletes or healthy individuals, or for health interventions.

## Materials and methods

A literature search protocol was constructed as described in Table 1. Databases relevant to health and exercise available at the University’s library, as the information source, were systematically searched through to 31^st^ of December 2020. The literature search and appraisal were conducted in four steps.

**Table 1:** Literature search protocol.

Databases and date range searched	Search field selections	Specific limitations	Number of items found
**EBSCO**	Default ‘Field’ for the first three sets of Booleans and search strings, and ‘All Text’ for the last set of Booleans and search strings (as detailed in Step 1)	Boolean/Phrase Apply equivalent subjects	ES = 1276
Jan.1950 –Dec.2020			VB = 166
		• AMED—Document Type: Journal Article	ND = 120
		• CINAHL—Research Article; Publication Type: Journal Article	
		• Health Business Elite—Publication Type: All	
		• Health Source (Nursing/Academic)–Publication Type: Academic Journal; Document Type: Article	
		• MEDLINE with Full Text—Publication Type: Journal Article	
		• APA PsycArticles—Document Type: Journal Article	
		• APA PsycInfo—Publication Type: Peer Reviewed Journal; Document Type: Journal Article	
		• Psychology and Behavioral Sciences Collections—Document Type: Article	
		• SPORTDiscus with Full Text—Publication Type: Academic Journal; Document Type: Article	
**ProQuest**	‘NOFT’ for the first three sets of Booleans and search strings, and ‘Anywhere’ for the last set of Boolean and search strings	Limit to: Peer reviewed	ES = 304
1/1/1950-31/12/2020		Source type: Scholarly journals	VB = 97
		Document Type: Article	ND = 40
**PubMed Central**	‘Abstract’ for the first two sets of Booleans and search strings, and ‘All Fields’ for the last two sets of Booleans and search strings		ES = 549
1/1/1950-31/12/2020			VB = 147
			ND = 66
**SCOPUS**	‘TITLE-ABS-KEY’ for the first three sets of Booleans and search strings, and ‘ALL’ for the last set of Boolean and search strings	Document Type: Article	ES = 1090
1959–2020		Source Type: Journal	VB = 236
			ND = 178
**Web of Science Core Collection**	‘ALL FIELDS’ for all Booleans and search strings	Document Types: Article	ES = 470
			VB = 135
1975–2020			ND = 131
**Total**			ES = 3689
			VB = 781
			ND = 535
			**Total = 5005**

Keys: ES: electric stimulation, VB: vibration, and ND: needling.

### Step 1—Searching databases

The following Booleans and search strings were used in the search: (“cross education” OR cross-education OR “cross transfer” OR cross-transfer OR cross training OR “cross-training” OR unilateral OR contralateral) AND (muscle OR muscular OR neuromuscular OR “motor function” OR “neural function”) AND (rehabilitation OR therapy OR treatment OR training) AND (“electrical stimulation” OR “electric stimulation” OR electromyostimulation OR “neuromuscular electrical stimulation” OR NMES). The last set of Booleans and search strings (after the AND in brackets) was replaced by (vibration OR vibratory) or (acupuncture OR electroacupuncture OR needling OR dry-needling) in the respective searches.

Table 1 shows the databases searched, the specific search strategies and limits, and the number of items found. The search outcomes were downloaded to EndNote (version X9.3.3) libraries and screened to remove duplicates, review articles, books and book sections, conference abstracts, and animal studies.

### Step 2—Screening for eligible studies

The EndNote library was then screened for eligible items in “Any Field”, using the words (or a part of a key word) and Booleans and search strings in the sequence of (1) “electr” (or “vibrat”, or “acup” or “needl” in the respective libraries), (2) “unilateral” or “contralateral” or “local” or “focal” or “cross”, and (3) “strength” or “force” or “torque” or “power” or “function”. The search was performed by one author and verified by third-party assistants to ensure the reproducibility of the search outcomes, and a 100% match between repeated searches was achieved.

### Step 3—Data charting and calibration

The eligible items identified in Step 2 were further screened against the following inclusion and exclusion criteria. The full text was reviewed if a decision could not be made from the title and abstract. One author screened all records. Another author also screened at least 50 randomly selected records in each of the ES, VB and ND areas, and compared the outcomes with the first author as a means of calibration. If there were discrepancies, they were verified by the authors to achieve 100% agreement.

#### Inclusion criteria

Investigations on human participants; empirical research; applied a unilateral peripheral neuromuscular stimulation; and assessed neuromuscular function of the stimulated side and/or the unstimulated contralateral side of the body, are eligible for inclusion. Articles written in languages other than English but with an abstract (or translation of the text) in English that presented information required in this scoping review were also eligible for inclusion.

#### Exclusion criteria

**Fig 1:**
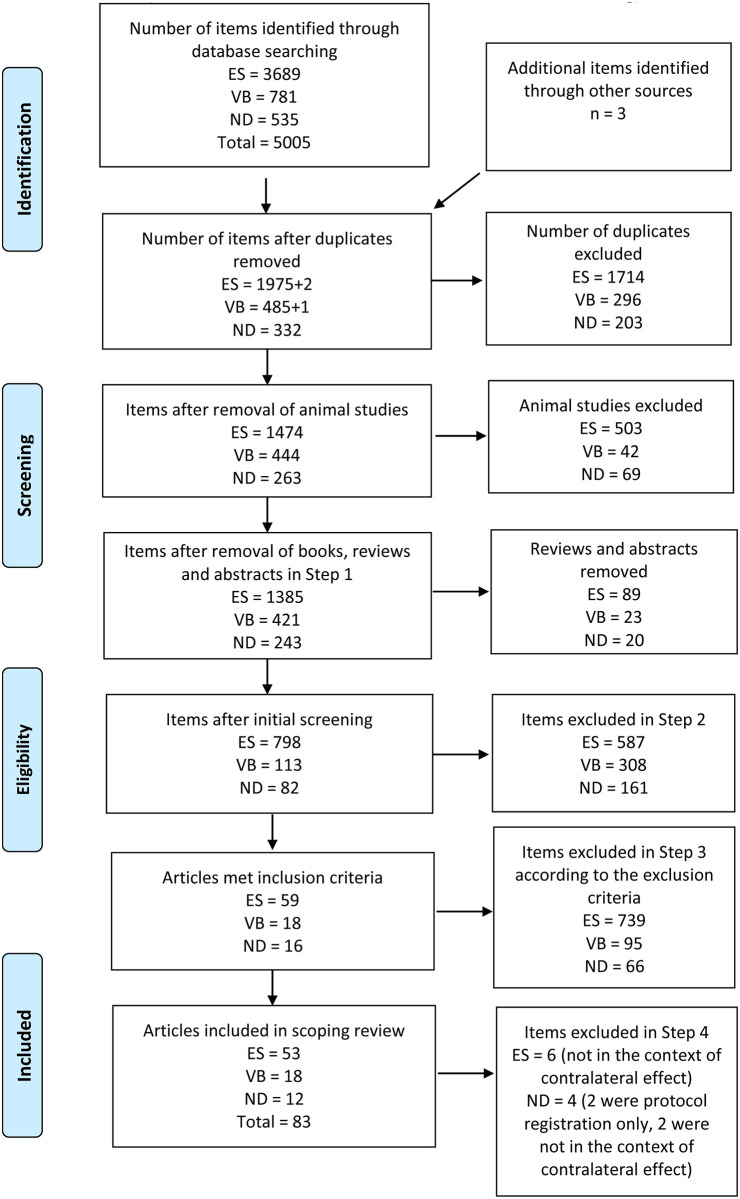
PRISMA flow diagram for database search outcomes. ES = electrical stimulation, VB = vibration, ND = needling.

Studies using animal models; review articles; conference abstracts; stimulation was only applied directly to the brain or spinal cord; and peripheral stimulation was applied on both sides of the body simultaneously, were excluded. Fig 1 summarises the search and screening results, following the PRISMA Extension for Scoping Review [37, 48].

### Step 4—Critical appraisal

The full text of each article eligible for inclusion was appraised according to the aim of the review.

## Results

The literature search resulted in a total of 5,005 items, of which 83 articles were identified as eligible for the scoping review, with 53 on ES, 18 on VB, and 12 on ND (Fig 1). Among these studies, there was one study [23] that included an ES and a VB group, three studies [24, 25, 41] that included both manual needling and electrical stimulation via needling groups, and one study [49] that included a manual needling and a vibration group.

The demographic information (year of publication, country and laboratory), contexts, participants, setting, and design of the reviewed articles are presented in Table 2 [12, 19–26, 41, 49–121].

**Table 2:** Demographic characteristics of the reviewed articles.

[Ref.] ID	Citation	Country/region, and the first affiliation of the first author	Context						Participants			Age	Gender			Setting		Design			
			Cross-education effect	Contralateral side as control	CNS plasticity	Clinical efficacy of unilateral intervention	Combined with other interventions	Did not address cross-education	Healthy	Patients with stroke or CNS disorders	Patients with post injury or operation	Age (year range or mean ± SD)	Female, number	Male, number	Not specified, number	Clinical	Laboratory	Randomised controlled	Convenience or case-matched control	Single group self-control	Single case study
[50]	Laughman et al., 1983	USA, Mayo Clinic and Mayo Foundation		*					*			21–39	30	28			*	*			
ES1																					
[51]	Singer, 1986	Australia, University of Western Australia				*					*	34.4±5.8		15			*			*	
ES2																					
[52]	Cabric et al., 1987	Yugoslavia, Split University	*						*			20–23		36			*	*			
ES3																					
[53]	Cannon et al., 1987	Canada, York University		*					*			N/A			23		*		*		
ES4																					
[54]	Lai, 1988	Australia, Curtin University of Technology	*						*			23.3–26.8	12	12			*	*			
ES5																					
[55]	Milner-Brown et al., 1988	USA, Children’s Hospital of San Francisco		*			*				*	17–62			10	*				*	
ES6																					
[56]	Gibson et al., 1989	UK, Princess Margaret Rose Orthopaedic Hospital				*		*			*	57–82			14		*		*		
ES7																					
[57]	Tachino et al., 1989	Japan, University of Kanazawa	*						*			stretched 19.7±1.1 shortened 19.5±1.2	20				*		*		
ES8																					
[58]	Abdel-Moty et al., 1994	USA, University of Miami		*							*	29–78	8	10		*				*	
ES9																					
[59]	Seib, et al., 1994	USA, University of Washington		*				*			*	19–73	4	6			*			*	
ES10																					
[60]	Paquet et al., 1996	Canada, McGill University			*		*	*	*			26±3			17		*			*	
ES11																					
[61]	Hollman et al., 1997	USA, University of Wisconsin-Madison		*				*	*			19–46	7	9			*			*	
ES12																					
[62]	Hortobágyi et al., 1999	USA, East Carolina University	*						*			24.8±4.5	32				*	*			
ES13																					
[63]	Lin, 2000	China (Taiwan), National Cheng-Kung University Hospital				*		*		*		29–75	1	3		*				*	
ES14																					
[64]	Zhou et al., 2002	Australia, Southern Cross University	*						*			22.6±3.0		30			*		*		
ES15																					
[65]	Han, et al., 2003	South Korea, Yeungnam University			*			*	*			20–38		8			*			*	
ES16																					
[66]	Marqueste et al., 2003	France, Universite´ de la Me´diterrane´e		*				*	*			26.1±2.5		7			*			*	
ES17																					
[67]	Stephen et al., 2003	USA, University of New Mexico			*		*		*			31–60	3	4			*			*	
ES18																					
[68]	Talbot et al., 2003	USA, The Johns Hopkins University		*				*			*	NMES 70.3±5.6			34	*		*			
ES19												control 70.8±4.9									
[69]	Lazcorreta et al., 2006	Spain, Universidad de Valencia	*						*			18–55		20			*	*			
ES20																					
[70]	Toca-Herrera et al., 2008	Spain, University of Valencia	*						*			25.8±1.3		36			*	*			
ES21																					
[71]	Yu et al., 2008	China, Tianjin University of Sport	*						*			18–30		30			*	*			
ES22																					
[12]	Bezerra et al., 2009	Australia, Southern Cross University	*				*		*			18–33		30			*	*			
ES23																					
[72]	Blickenstorfer et al., 2009	Switzerland, University Hospital Zurich			*				*			31.3±7.9	8	7			*			*	
ES24																					
[73]	Francis et al., 2009	UK, University of Norttingham			*			*	*			30±7	5	9			*			*	
ES25																					
[74]	Pietrosimone et al., 2010	USA, University of Toledo				*					*	TENS 60.3±11.9 placebo 58.7±12.2 control 58.3±11.8	20	13		*		*			
ES26																					
[75]	Suetta et al., 2010	Denmark, University of Copenhagen		*				*			*	60–79	14	14			*	*			
ES27																					
[76]	Pittaccio et al., 2011	Italy, Unita`di Lecco			*			*	*			29±7	2	5			*			*	
ES28																					
[77]	Sariyildiz et al., 2011	Turkey, Vakif Gureba Training and Research Hospital	*				*		*			29.6±5.7		23			*	*			
ES29																					
[78]	Joa et al., 2012	South Korea, Pusan National University			*			*	*			28±3	4	19			*			*	
ES30																					
[79]	Lagerquist et al., 2012	Canada, Northern Alberta Institute of Technology			*				*			22–44	3	7			*			*	
ES31																					
[80]	Popa et al., 2012	Romania, University of Medicine and Pharmacy Gr. T. Popa	*							*		35–85	21	20		*			*		
ES32																					
[81]	Einhorn et al., 2013	USA, New York University			*			*	*			23–42	5	8			*			*	
ES33																					
[82]	Liu et al., 2013	China, Chinese PLA General Hospital			*			*		*		40		1			*				*
ES34																					
[83]	Popa et al., 2013	Romania, University of Medicine and Pharmacy Gr. T. Popa				*				*		patients 67.3±10.3 healthy 56.0±14.2	10	10		*			*		
ES35																					
[20]	Onigbinde et al., 2014	Nigeria, Obafemi Awolowo University	*						*			23.9±2.1			50		*			*	
ES36																					
[84]	Dietz et al., 2015	Switzerland, Balgrist University Hospital			*			*	*			25–32	15	17			*			*	
ES37																					
[85]	Lepley et al., 2015	USA, University of Kentucky		*				*			*	14–30	13	23		*			*		
ES38																					
[86]	Muthalib et al., 2015	France, University of Montpellier			*			*	*			39.2±13.0		9			*			*	
ES39																					
[87]	Andrade et al., 2016	Brazil, Federal University of Paraná		*					*			22.5±1.9		7			*			*	
ES40																					
[88]	Schrafl-Altermatt et al., 2016	Switzerland, Balgrist University Hospital				*		*		*		38–60	4	11		*			*		
ES41																					
[89]	Suzuki et al., 2016	Japan, Tokyo Gakugei University			*			*	*			20–35			7		*			*	
ES42																					
[90]	Gueugneau et al., 2017	France, Institut National de la Santé et de la Recherche Médicale			*				*			19–45		10			*			*	
ES43																					
[19]	Kadri et al., 2017	Algeria, Université Badji Mokhtar Annaba	*						*			21–31		36			*	*			
ES44																					
[91]	Cattagni et al., 2018	France, Université Bourgogne Franche-Comté	*						*			30±7		20			*			*	
ES45																					
[92]	Jang, 2018	South Korea, Yeungnam University		*				*	*			21–33	8	5			*			*	
ES46																					
[93]	Kwong et al., 2018	China (Hong Kong), Hong Kong Polytechnic University				*	*	*		*		55–85			80	*		*			
ES47																					
[94]	Yang et al., 2018	China, Soochow University				*		*		*		control 67.2±10.7 FMS 63.7±15.1	7	23		*			*		
ES48																					
[95]	Sun et al., 2019	Canada, University of Victoria			*		*		*			26.7±4.9			13		*			*	
ES49																					
[96]	Barss et al., 2020	Canada, University of Victoria	*						*			TRAIN 22.1±4.1 STIM 23.3±2.8 T+S 22.4±2.8	18	9			*	*			
ES50																					
[97]	Benito-Martínez et al., 2020	Spain, Comillas Pontifical University	*						*			18–26	25	20			*			*	
ES51																					
[98]	Segers et al., 2020	Belgium, KU Leuven		*							*	60±15	22	25		*				*	
ES52																					
[99]	Yurdakul et al., 2020	Turkey, Bezmialem Vakif University	*							*		43–89	17	13		*		*			
ES53																					
Sum	27 in 1983–2010	USA 11, Canada 5, Australia 4, France 4, China (mainland) 3, South Korea 3, Spain 3, Switzerland 3, Japan 2, Romania 2, Turkey 2, UK 2, Algeria 1, Belgium 1, Brazil 1, Denmark 1, China (Hong Kong) 1, Italy 1, Nigeria 1, China (Taiwan) 1, Yugoslavia 1	17	13	15	8	7	22	35	8	10	14–89	338	648	248	13	40	16	9	27	1
	26 in 2011–2020																				
			Cross-education effect	Contralateral side as control	CNS plasticity	Clinical efficacy of unilateral intervention	Combined with other interventions	Did not address cross-education	Healthy	Patients with stroke or CNS disorders	Patients with post injury or operation	Age (year range or mean ± SD)	Female, number	Male, number	Not specified, number	Clinical	Laboratory	Randomised controlled	Convenience or case-matched control	Single group self-control	Single case study
[100]	Jackson et al., 2003	UK, South Bank University	*						*			26±2		10			*			*	
VB1																					
[101]	Christova et al., 2010	Austria, Medical University of Graz			*		*		*			26.6±6.1	8	4			*			*	
VB2																					
[102]	Fowler et al., 2010	Turkey, Ege University			*			*	*			18–28		22			*			*	
VB3																					
[103]	Couto et al., 2012	Brazil, Load Assessment Laboratory—CENESP / UFMG	*				*		*			24.5±4.2		29			*	*			
VB4																					
[104]	Dickerson et al., 2012	USA, University of Puget Sound				*		*	*			22–32	19	11			*	*			
VB5																					
[21]	Goodwill et al., 2012	Australia, Deakin University	*						*			18–35	12	9			*	*			
VB6																					
[105]	Karacan et al., 2012	Turkey, Vakıf Gureba Training and Research Hospital	*						*			20–52	33	57			*			*	
VB7																					
[106]	Lin et al., 2012	China (Taiwan), National Cheng Kung University				*		*		*		female 59.6±16.0 male 61.1±15.3	9	26		*				*	
VB8																					
[107]	Lapole et al., 2013	France, Université de Lyon, Université Jean Monnet Saint-Etienne	*						*			22.2±2.7			11		*			*	
VB9																					
[108]	Marín et al., 2014	Spain, European University Miguel de Cervantes	*						*			20.8±1.2		17			*			*	
VB10																					
[109]	Souron et al., 2017	France, Université de Lyon, Université Jean Monnet Saint-Etienne	*						*			20±1	24	20			*	*			
VB11																					
[110]	García-Gutiérrez et al., 2018	Spain, European University Miguel de Cervantes	*						*			female 19.5±7.2 male 21.8±2.7	19	19			*			*	
VB12																					
[23]	Minetto et al., 2018	Italy, University of Turin	*						*			27.8±5.8		11			*			*	
VB13																					
[22]	Li et al., 2019a	China, Tsinghua University			*			*	*			26±0.6		20			*			*	
VB14																					
[112]	Li et al., 2019b	China, Tsinghua University			*			*		*		18–75	2	19			*		*		
VB15												healthy 24±4.3 patients 53±2.2									
[111]	Wang et al., 2019	China (Taiwan), National Taiwan University				*		*			*	patients 25–27 control 29–60	6	26			*		*		
VB16																					
[113]	Aydin et al., 2020	Turkey, Istanbul Physical Medicine Rehabilitation Training and Research Hospital	*						*			32.3±6.9		42			*	*			
VB17																					
[114]	Delkhoush et al., 2020	Iran, Semnan University of Medical Sciences	*						*			20–35	14	14			*	*			
VB18																					
Sum	3 in 2003–2010	Turkey 3, China (mainland) 2, France 2, Spain 2, China (Taiwan) 2, Australia 1, Austria 1, Brazil 1, Iran 1, Italy 1, UK 1, USA 1	11	0	4	3	2	6	15	2	1	18–75	146	346	11	1	17	6	2	10	0
	15 in 2011–2020																				
			Cross-education effect	Contralateral side as control	CNS plasticity	Clinical efficacy of unilateral intervention	Combined with other interventions	Did not address cross-education	Healthy	Patients with stroke or CNS disorders	Patients with post injury or operation	Age (year range or mean ± SD)	Female, number	Male, number	Not specified, number	Clinical	Laboratory	Randomised controlled	Convenience or case-matched control	Single group self-control	Single case study
[49]	Takakura et al., 1992	Japan, Japan Central Acupuncture and Moxibustion College			*			*	*			20–47			66		*			*	
ND1																					
[115]	Audette et al., 2004	USA, Harvard Medical School				*					*	19–71			13		*		*		
ND2																					
[41]	Huang et al., 2007	China, Tianjin University of Sport	*						*			20.9±3.0		30			*	*			
ND3																					
[24]	Zhou et al., 2012	Australia, Southern Cross University	*						*			20.6±2.2		43			*	*			
ND4																					
[116]	Zanin et al., 2014	Brazil, University of Sa˜o Paulo				*		*	*			18–30			52		*	*			
ND5																					
[117]	Chen et al., 2015	China, Beijing University of Chinese Medicine			*			*		*		50–76		6		*				*	
ND6																					
[25]	Huang et al., 2015	China, Tianjin University of Sport	*						*			19–27		50			*	*			
ND7																					
[26]	de Souza et al., 2016	Brazil, University of São Paulo				*		*	*			18–30	29	9			*	*			
ND8																					
[118]	Bandeira et al., 2019	Brazil, Universidade Federal do Rio Grande do Sul			*				*			20–55		15			*	*			
ND9																					
[119]	He et al., 2019	China, Fujian University of Traditional Chinese Medicine			*			*	*			18–34	8	10			*	*			
ND10																					
[120]	Chen et al., 2020	China, Guangzhou University of Chinese Medicine			*			*		*		44–68	3	7			*			*	
ND11																					
[121]	Garcia-de-Miguel et al., 2020	Spain, University of Alcalá				*		*			*	DN 25.5±8.5 PENS 24.1±9.4	28	16		*		*			
ND12																					
Sum	3 in 1992–2010	China (mainland) 5, Brazil 3, USA 1, Australia 1, Japan 1, Spain 1	3	0	5	4	0	7	8	2	2	18–76	68	186	131	2	10	8	1	3	0
	9 in 2011–2020																				
			Cross-education effect	Contralateral side as control	CNS plasticity	Clinical efficacy of unilateral intervention	Combined with other interventions	Did not address cross-education	Healthy	Patients with stroke or CNS disorders	Patients with post injury or operation	Age (year range or mean ± SD)	Female, number	Male, number	Not specified, number	Clinical	Laboratory	Randomised controlled	Convenience or case-matched control	Single group self-control	Single case study
Total	33 in 1983–2010	USA 13, China (mainland) 11, France 6, Spain 6, Australia 5, Brazil 5, Canada 5, Turkey 5, Japan 3, South Korea 3, Switzerland 3, China (Taiwan) 3, UK 3, Italy 2, Romania 2, 1 each from Algeria, Austria, Belgium, Denmark, China (Hong Kong), Iran, Nigeria and Yugoslavia	31	13	24	15	9	35	58	12	13	14–89	552,	1190,	390	16	67	30	12	40	1
83	50 in 2011–2020											or group mean	2F only	26M only							
												19.5–70.8									

### Context of the studies

Amongst the 83 articles reviewed, 31 (37.3%) claimed that their studies examined cross-education, 13 (15.7%) investigated the effects of unilateral stimulation with the contralateral side as control, 24 (28.9%) examined the effects of unilateral stimulation on CNS activities or plasticity (e.g. assessment by fMRI, fNIRs, TMS, MEP or EEG, etc.), and 15 (18.1%) examined clinical efficacy of unilateral interventions. Nine (10.8%) applied peripheral stimulation in combination with other types of interventions, and 35 (42.2%) applied unilateral stimulation but did not mention cross-education at all (Table 2).

### Participants

The majority of the articles (58/83, 69.9%) reported effects of various interventions on healthy participants, 25 (30.1%) studies were on patients, including those with stroke (10, 12.0%), pain (3, 3.6%), injuries or surgeries (5, 6.0%), arthritis or muscle dystrophy (4, 4.8%), Parkinson’s disease (2, 2.4%) or critically ill (1, 1.2%) (Table 2).

### Design of the studies

Thirty (30/83, 36.1%) studies claimed that they utilised a randomised, controlled design; and among them six studies investigated on patients [68, 74, 75, 93, 99, 121], whilst only one study claimed to be in the context of cross-education [99]. There were 12 (14.5%) studies utilised non-randomised or case-matched controls, with eight were on patients [56, 80, 83, 88, 94, 111, 112, 115]. There were 40 (48.2%) studies used a single group, self-controlled design, with nine investigated on patients [55, 58, 59, 63, 85, 98, 106, 117, 120]; while there was one (1.2%) single case study on a patient [82]. Sixteen (16, 19.3%) studies were conducted in clinical settings and 67 (80.7%) were in laboratory settings (Table 2).

The intervention protocols, including the muscles and nerves stimulated, outcome measures (muscle strength, neuromuscular function, muscle activation, muscle size, and CNS responses), and statistical analyses used in the studies are presented in Table 3.

**Table 3:** Intervention protocols and outcome measures.

[Ref.] ID	Citation	Muscle or nerve stimulated	Stim. side	Intervention protocols					Muscle function			Muscle activation		Muscle size		CNS	Statistical analysis			
			A: affected; W: weaker; D: dominant; ND: non-dominant; R: right; L: left; N: not stated	Acute effect	Chronic effect: sessions per week x number of weeks = total number of sessions	Detailed stimulation parameters	Static or isometric contraction	Dynamic/isotonic, eccentric	Muscle strength of treated side	Muscle strength of contralateral side	Other functional assessments	Twitch interpolation	EMG	Imaging (MRI, CT, Ultrasound, fibre type)	Girth	EEG, fMRI, TMS-MEP, fNIR, reflexes	P value based	Effect size reported	Assumptions checked	Sample size justified
[50]	Laughman et al., 1983	Knee extensors	R		5 x 5 = 25	*	*		*	*							*			
ES1																				
[51]	Singer, 1986	Knee extensors	A		7 x 4 = 28	*	*		*	*			*	*			*			
ES2																				
[52]	Cabric et al., 1987	Ankle plantar flexors	N		3 x 7 = 21	*	*		*	*					*		*			
ES3																				
[53]	Cannon et al., 1987	Adductor pollicis	R		3 x 5 = 15	*	*		*	*	*		*				*			
ES4																				
[54]	Lai, 1988	Knee extensors	L		5 x 3 = 15	*	*		*	*							*			
ES5																				
[55]	Milner-Brown et al., 1988	Ankle dorsiflexors and knee extensors	A		5 x 56 = 280	*		ǁ †	*	*							*			
ES6																				
[56]	Gibson et al., 1989	Knee extensors	A		4 x 7 = 28	*	*		*	*				*FT			*			
ES7																				
[57]	Tachino et al., 1989	Ankle dorsiflexors	L		4 x 6 = 24	*	*		*	*	*						*			
ES8																				
[58]	Abdel-Moty et al., 1994	Knee extensors and ankle dorsiflexors	A		5 x 6 = 30	*	*†		*	*							*			
ES9																				
[59]	Seib, et al., 1994	Ankle dorsiflexors	A	*		ǂ	ǂ				*						*			
ES10																				
[60]	Paquet et al., 1996	Ankle dorsiflexors and knee extensors	R	*		*	*						*			*	*			
ES11																				
[61]	Hollman et al., 1997	Handgrip muscles	L	*		*	*				*					*	*			
ES12																				
[62]	Hortobágyi et al., 1999	Knee extensors	L		4 x 6 = 24	*		§	*	*			*				*			
ES13																				
[63]	Lin, 2000	Ulnar nerve and extensor digitorum communis	A	*		*		ǁ			*		*				*			
ES14																				
[64]	Zhou et al., 2002	Knee extensors	D		3 x 4 = 12	*	*		*	*			*				*			
ES15																				
[65]	Han, et al., 2003	Wrist extensors	D	*		*	*									*	*			
ES16																				
[66]	Marqueste et al., 2003	Knee extensors	W		5 x 6 = 30	*	*		*	*			*				*			
ES17																				
[67]	Stephen et al., 2003	Elbow flexors and the deltoid	RL	*		ǂ	ǂ †						*			*	*			
ES18																				
[68]	Talbot et al., 2003	Knee extensors	A		3 x 12 = 36	*	*		*	*	*						*		*	
ES19																				
[69]	Lazcorreta et al., 2006	Knee extensors	R	*		*	*			*							*			
ES20																				
[70]	Toca-Herrera et al., 2008	Knee extensors	ND	*		*	*		*	*			*				*			
ES21																				
[71]	Yu et al., 2008	Ankle dorsiflexors	R		4 x 6 = 24	*	*		*	*		*					*			
ES22																				
[12]	Bezerra et al., 2009	Knee extensors	R		3 x 6 = 18	*	*†		*	*				*			*			
ES23																				
[72]	Blickenstorfer et al., 2009	Wrist flexors and extensors	N	*		*		ǁ								*	*			
ES24																				
[73]	Francis et al., 2009	Common peroneal nerve, ankle dorsiflexors	R	*		*	*						*			*	*			
ES25																				
[74]	Pietrosimone et al., 2010	Knee extensors	A		3 x 4 = 12	*		ǁ		*		*					*	*		
ES26																				
[75]	Suetta et al., 2010	Knee extensors	A		3 x 12 = 36	ǂ	ǂ							*FT			*		*	
ES27																				
[76]	Pittaccio et al., 2011	Common peroneal nerve, ankle dorsiflexors	RL	*		*	*						*			*	*		*	
ES28																				
[77]	Sariyildiz et al., 2011	Wrist flexors	R		5 x 6 = 30	*		† §	*	*							*		*	
ES29																				
[78]	Joa et al., 2012	Wrist extensors	D	*		*		ǁ								*	*			
ES30																				
[79]	Lagerquist et al., 2012	Tibia nerve, ankle plantar flexors	R	*		*	*		*	*			*			*	*		*	
ES31																				
[80]	Popa et al., 2012	Common peroneal nerve and radial nerve	A		10	*	ǂ				*						*			
ES32																				
[81]	Einhorn et al., 2013	Median nerve, flexor carpi radialis, elbow flexors and extensors	LT	*		*	ǂ						*			*	*			
ES33																				
[82]	Liu et al., 2013	Hand muscles	A		5 x 4 = 20	*		ǁ			*					*				
ES34																				
[83]	Popa et al., 2013	Radial nerve	A		10	*	ǂ				*		*			*	*			
ES35																				
[20]	Onigbinde et al., 2014	Knee extensors	R		2 x 8 = 16	*	*		*	*							*			
ES36																				
[84]	Dietz et al., 2015	Ulnar nerve, wrist flexors and extensors	D	*		*		ǁ †			*		*			*	*			
ES37																				
[85]	Lepley et al., 2015	Knee extensors	A		2 x 6 = 12	*	*	§	*	*	*						*	*		*
ES38																				
[86]	Muthalib et al., 2015	Wrist extensors	R	*		*		ǁ								*	*		*	
ES39																				
[87]	Andrade et al., 2016	Ankle plantar flexors	N		3 x 6 = 18	*	*		*	*				*			*	*		
ES40																				
[88]	Schrafl-Altermatt et al., 2016	Ulnar nerve, forearm muscles	A	*		*		ǁ					*			*	*			
ES41																				
[89]	Suzuki et al., 2016	Ankle plantar flexors and dorsi flexors	R	*		*		ǁ					*			*	*		*	
ES42																				
[90]	Gueugneau et al., 2017	Flexor carpi radialis	D	*		*	*		*	*			*			*	*			
ES43																				
[19]	Kadri et al., 2017	Knee extensors	D		3 x 8 = 24	*	*		*	*	*						*		*	
ES44																				
[91]	Cattagni et al., 2018	Knee extensors	R	*		*	*			*		*	*				*	*	*	
ES45																				
[92]	Jang, 2018	Finger extensors	D		7 x 2 = 14	*		ǁ	*	*	*						*			
ES46																				
[93]	Kwong et al., 2018	Peroneal nerve, ankle dorsiflexors and plantar flexors, and knee extensors and flexors	A		2 x 10 = 20	*		ǁ †	*	*	*						*			*
ES47																				
[94]	Yang et al., 2018	Supraspinatus and deltoid	A		5 x 4 = 20	*		ǁ			*			*			*			
ES48																				
[95]	Sun et al., 2019	Extensor carpi radialis and flexor carpi radialis	ND	*		ǂ	ǂ †						*			*	*			
ES49																				
[96]	Barss et al., 2020	Wrist extensors	R		3 x 5 = 15	*	*		*	*			*			*	*	*	*	
ES50																				
[97]	Benito-Martínez et al., 2020	Knee extensors	D&ND	*		*	ǂ									*	*		*	*
ES51																				
[98]	Segers et al., 2020	Knee extensors	RD		7 x 1 = 7	*	ǂ		*	*				*			*	*	*	*
ES52																				
[99]	Yurdakul et al., 2020	Wrist flexors	UA		5 x 6 = 30	*	ǂ		*	*	*						*	*	*	
ES53																				
Sum		Knee extensors 23	A16	22	31 duration	49	28	ǁ13	28	31	16	3	21	7	1	20	52	7	13	4
		Knee flexors 1	UA1			ǂ4	ǂ10	§3												
		Dorsiflexors 10	LT1		2–56 weeks,		†4	†4												
		Plantar flexors 5	D7		10–280 sessions															
		Elbow flexors 2	ND2																	
		Elbow extensors 1	D&ND1																	
		Deltoid 2	N3																	
		Wrist flexors 8	R14																	
		Wrist extensors 9	L4																	
		Hand muscles 6	RD1																	
		Paraspinal 1	RL2																	
		Nerves 10	W1																	
		Muscles examined	A: affected; D: dominant; ND: non-dominant; R: right; L: left; N: not stated	Acute effect	Chronic effect: sessions per week x number of weeks = total number of sessions	Detailed stimulation parameters	Static or isometric contraction	Dynamic/isotonic, eccentric	Muscle strength of treated side	Muscle strength of contralateral side	Other functional assessments	Twitch interpolation	EMG	Imaging (MRI, CT, Ultrasound, fibre type)	Girth	EEG, fMRI, TMS-MEP, fNIR, reflexes	P value based	Effect size reported	Assumptions checked	Sample size justified
[100]	Jackson et al., 2003	Knee extensors	R	*		*	*		*	*			*				*			
VB1																				
[101]	Christova et al., 2010	First dorsal interosseous	R	*		*		ǁ †								*	*			
VB2																				
[102]	Fowler et al., 2010	Flexor digitorum sublimis	D	*		*		ǁ					*			*	*			
VB3																				
[103]	Couto et al., 2012	Elbow extensors and flexors	N		4 weeks	*	*†		*	*			*				*			
VB4																				
[104]	Dickerson et al., 2012	Knee extensors and flexors	R	*		*	*		*	*							*		*	
VB5																				
[21]	Goodwill et al., 2012	Knee extensors	D		3 x 3 = 9	*		ǁ	*	*			*	*		*	*	*	*	*
VB6																				
[105]	Karacan et al., 2012	Wrist flexors	R	*		*	*				*		*				*		*	
VB7																				
[106]	Lin et al., 2012	Hip flexors, knee extensors and ankle dorsiflexors	RL	*		*		ǁ	*	*	*						*		*	
VB8																				
[107]	Lapole et al., 2013	Ankle plantar flexors	R		5 x 2 = 10	*	*		*	*			*			*	*		*	
VB9																				
[108]	Marín et al., 2014	Knee extensors and ankle plantar flexors	D	*		*	*				*		*				*	*	*	
VB10																				
[109]	Souron et al., 2017	Ankle dorsiflexors	R		3 x 8 = 24	*	*		*	*			*			*	*		*	*
VB11																				
[110]	García-Gutiérrez et al., 2018	Ankle plantar flexors and dorsiflexors	D	*		*	*		*	*							*	*	*	
VB12																				
[23]	Minetto et al., 2018	Knee extensors	R	*		*	*			*		*	*				*		*	
VB13																				
[22]	Li et al., 2019a	Elbow flexors	L	*		*	*									*	*		*	
VB14																				
[112]	Li et al., 2019b	Ankle plantar flexors	A	*		*	*									*	*	*	*	
VB15																				
[111]	Wang et al., 2019	Ankle plantar flexors	A		7 x 4 = 28	*	*				*						*			*
VB16																				
[113]	Aydin et al., 2020	Knee extensors	D		5 x 4 = 20	*			*	*			*			*	*		*	*
VB17																				
[114]	Delkhoush et al., 2020	Hand muscles	D	*		*	*	ǁ		*			*				*		*	*
VB18																				
Sum		Knee extensors 7	A2	12	6	18	13	5ǁ	9	11	4	1	11	1	0	8	18	4	13	5
		Knee flexors 1	D6		duration		†1	†1												
		Dorsiflexors 3	N1		2–8 weeks															
		Plantar flexors 5	R7		9–28 sessions															
		Elbow flexors 2	L1																	
		Elbow extensors 1	RL1																	
		Wrist flexors 1																		
		Hand muscles 3																		
		Hip flexors 1																		
		Muscles received needling	A: affected; D: dominant; R: right; L: left; UA: unaffected	Acute effect	Chronic effect: sessions per week x number of weeks = total number of sessions	Detailed stimulation parameters	Static or isometric contraction	E: needling with electrical stimulation, V: with vibration	Muscle strength of treated side	Muscle strength of contralateral side	Other functional assessments	Twitch interpolation	EMG	Imaging (MRI, CT, Ultrasound, fibre type)	Girth	EEG, fMRI, TMS-MEP, fNIR, reflexes	P value based	Effect size reported	Assumptions checked	Sample size justified
[49]	Takakura et al., 1992	Hoko point on hand	R	*		*	*	V	*	*						*	*			
ND1																				
[115]	Audette et al., 2004	Trapezius and paraspinal muscles	R	*		*	*						*				*			
ND2																				
[41]	Huang et al., 2007	ST36 and ST39, on leg	R		3 x 4 = 12	*	*	E	*	*							*			*
ND3																				
[24]	Zhou et al., 2012	ST36 and ST39, on leg	R		3 x 6 = 18	*	*	E	*	*							*			*
ND4																				
[116]	Zanin et al., 2014	HT3, HT4 on arm	D	*		*	*		*	*			*				*		*	
ND5																				
[117]	Chen et al., 2015	GB34 on leg	A	*		*	*									*	*			
ND6																				
[25]	Huang et al., 2015	ST36 and ST39, on leg	R		3 x 8 = 24	*	*	E	*	*		*					*			*
ND7																				
[26]	de Souza et al., 2016	SI14 and SI8 on arm	R	*		*	*		*	*			*				*		*	
ND8																				
[118]	Bandeira et al., 2019	Accessory spinal nerve	R	*		*	ǂ	E								*	*		*	
ND9																				
[119]	He et al., 2019	LI11 and TB5 on arm	L	*		*	*									*	*			
ND10																				
[120]	Chen et al., 2020	LI11 on arm and ST36 on leg	UA	*		*	ǂ									*	*			
ND11																				
[121]	Garcia-de-Miguel et al., 2020	Trigger point on neck	A	*		*	*	E	*		*						*	*	*	*
ND12																				
Sum		Arm 5	A2	9	3	12	10	E5	7	6	1	1	3	0	0	5	12	1	4	4
		Leg 5	UA1		duration		ǂ2	V1												
		Neck 1	D1		4–8 weeks															
		Nerve 1	R7		12–24 sessions															
		Paraspinal 1	L1																	
Total		Knee extensors 30	A20	43	40	78	51	ǁ18	44	48	21	5	35	8	1	33	82	12	30	13
		Knee flexors 2	UA2		duration	ǂ4	ǂ12	§3												
		Dorsiflexors 13	LT1		2–56 weeks		†5	E5												
		Plantar flexors 10	D14					V1												
		Elbow flexors 4	ND2		9–280 sessions			†5												
		Deltoid 2	D&ND1																	
		Elbow extensors 2	N4																	
		Wrist flexors 9	R28																	
		Wrist extensors 9	L6																	
		Hand muscles 9	RD1																	
		Hip flexors 1	RL3																	
		Paraspinal/neck 3	W1																	
		Arm 5																		
		Leg 5																		
		Nerve 11																		

Keys: A = affected side; CNS = central nervous system; CT = computed tomography; D = dominant side; E = electrical needling; EMG = electromyography; fMRI = functional MRI; fNIR = functional near infrared spectroscopy; FT: muscle fibre type; L = left side; LT = low threshold side; MEG = magnetoencephalography; MEP = motor evoked potential; ND = non-dominant side; N = not specified; R = right side; RD: randomly selected side; RL = right and left side alternatively; TMS = transcranial magnetic stimulation; UA = unaffected side; V = needling with vibration; W = weaker side;

^ǂ^ = not clearly explained or not relevant;

^†^ = stimulation combined with other intervention;

^ǁ^ = dynamic/isotonic;

^§^ = eccentric.

### Muscles and/or nerves stimulated

Many of the 83 studies involved stimulation of more than one muscle, or stimulation on nerves. For those stimulating one or more muscles, each muscle was recorded to one count. For the studies that applied needling to multiply points on a limb, each arm or leg was recorded as one count. There was a total of 104 counts of muscles or limbs. The knee extensors were most frequently investigated with 30 counts (30/104 = 28.8%), followed by 13 (12.5%) studies on ankle dorsiflexors, 10 (9.6%) on ankle plantar flexors, nine (8.7%) on each of the wrist flexors, wrist extensors and hand muscles, five (4.8%) on each of the arm and leg, four (4.2%) on the elbow flexors, two (1.9%) on each of the elbow extensors, deltoid, and knee flexors, three (2.9%) on paraspinal or neck muscles, and one (1.0%) on hip flexors. There were 11 (11/83 = 13.3%) studies that applied stimulation to nerves, including four on the peroneal nerve, three on the ulnar nerve, one each on the median nerve, radial nerve, tibia nerve, and accessory spinal nerve. Five of the 12 needling studies included one or more groups that applied electrical stimulation via the needles.

There were 21 studies (21/83, 25.3%) applied stimulation on the affected or weaker side in patients, 14 studies (16.9%) stated that the stimulation was on the dominant side, two (2.4%) on the non-dominant side, 28 (33.7%) on the right side (28, 33.7%), six (7.2%) on the left side, three (3.6%) on the right and left side alternately, and four (4.8%) did not state the side of stimulation (Table 3).

### Duration of the intervention

There were 40 articles (48.2%) that reported the effects of chronic peripheral stimulation (training), with 31, six and three applying ES, VB or ND, respectively; and 43 (51.8%) that investigated the acute effects, with 22, 12 and nine applying ES, VB or ND, respectively. Among the 40 studies that used chronic stimulation, the typical protocols involved 2–5 sessions per week (except for one ES study [80] that applied 10 sessions per week, and three ES studies [51, 92, 98] and one VB study [111] that trained for seven sessions per week), for 1–12 weeks with a total of 9–36 training sessions (with exception of one ES study [55] that lasted 56 weeks and involved 280 sessions) (Table 3).

### Types of muscle activity

Among the 83 studies, 51 (61.4%) induced or performed isometric contraction or participants held a static position during the intervention (28 under ES, 13 under VB, and 10 under ND); 21 (25.3%) induced or performed dynamic contraction (isotonic, concentric, eccentric, or joint movement; 16 under ES, five under VB), with one of them utilising both static and dynamic contractions [85]; and 12 (14.5%) did not specify the type of contraction or was deemed irrelevant (10 under ES and two under ND). There were 10 (12.0%) studies that employed peripheral stimulation combined with other types of interventions (e.g. voluntary muscle contraction) (Table 3).

### Stimulation properties

Most of the studies adequately described the stimulation parameters, while some did not describe the methods in detail. For example, among the 53 ES studies, 15 did not report the wave shape of the stimulation pulse, and one did not report stimulation frequency. Typically, ES studies employed stimulation frequencies between 20 and 300 Hz (with exception of two studies that used 2000 Hz and 2500 Hz respectively, and two studies used under 10 Hz), pulse width 50–500 μS (with exception of nine studies that reported 1.0–2.5 ms), biphasic symmetrical rectangular waves (13), rectangular/square waves (8), biphasic waves (8), monophasic wave (4), sine/alternate waves (3) or mixed wave types (1). For the 18 vibration studies, vibration frequency of 8–300 Hz and amplitude of 0.5–6.0 mm were used. For the 12 needling studies, nine reported regular hand manipulation of the needles during a session, and seven reported the depth of needle insertion.

### Assessments of intervention outcomes

Just under one-half of the studies (39, 47.0%) assessed muscle strength changes in response to the interventions for both the stimulated and unstimulated sides. In contrast, five studies (6.0%) only reported muscle strength changes for the contralateral (not directly stimulated) side of the body. There were 34 (41.0%) studies that did not use muscle strength as the major outcome measure but assessed other neuromuscular functions, such as CNS responses (31 used EEG, fMRI, TMS, fNIRs, or reflexes, etc.) or other functional responses. Thirty-five studies measured EMG changes, with most combined with other measurements such as strength, while five studies measured EMG only as the major outcome indicator [105, 108, 115]. Five studies assessed muscle activation using the twitch interpolation technique [23, 25, 71, 74, 91]. One study measured muscle girth change [52], seven studies utilised medical imaging methods, such as MRI, CT or ultrasound [12, 21, 51, 87, 98] or muscle fibre typing [56, 75] to determine muscle morphological changes (Table 3).

### Statistical analysis

The majority of the studies (82, 98.8%) employed statistical analyses that were P value based, and 12 studies (14.5%) reported the effect size (but did not necessarily employ a magnitude-based assessment). Among the 82 studies, 13 (15.8%) reported statistical justification for the sample size in their studies; and among the 45 studies that employed ANOVA or GLM analysis, 30 (30/45, 66.7%) reported assessment of sample normality against the assumptions of the method. Most of the studies that reported sample distribution (28/30) or justification of sample size (12/13) were publish after year 2010.

### Major findings from the studies on cross-education

Among the 83 studies, 31 (37.3%) claimed that their aim was to examine the cross-education effects, with 10 studies examining acute effects of unilateral stimulation and 21 studies investigating the chronic effects of repeated unilateral stimulation. The aims, major findings, strength of the research design, and limitations as stated by the authors are summarised in Table 4 [12, 19–21, 23–25, 41, 52, 54, 57, 62, 64, 69–71, 77, 80, 91, 96, 97, 99, 100, 103, 105, 107–110, 113, 114].

**Table 4:** The major characteristics of the studies on cross-education.

[Ref.] ID	Citation	Aim, context and method	Major findings	Characteristics in design	Limitations acknowledged in the article
**Acute effect**					
[69] ES20	Lazcorreta et al. 2006	To investigate the acute effect of unilateral NMES on the right quadriceps femoris on the contraction force of the left quadriceps, and the importance of the crossed extension reflex in cross-training effect in healthy men. Participants received NMES with the pulse width of 100 μs, frequency of 100 Hz and intensity of maximum tolerance for 1 min.	The maximal isometric knee extension force of the left leg was significantly increased after the right quadriceps received the NMES, while the control group showed no change in contraction force.	Randomised, controlled trial.	Not stated.
[70] ES21	Toca-Herrera et al. 2008	To investigate the acute effect of EMS on the rectus femoris of the non-dominant leg on isometric MVC, EMG and MMG of the dominant leg in healthy men. Participants received EMS with the pulse width of 300 μs, frequency of 100 Hz and intensity of maximum tolerance for 10 min (30 contractions).	The isometric knee extension strength of the dominant leg significantly increased in response to the contralateral stimulation; EMG of the agonist muscle increased, and that of the antagonist muscle decreased, while no change was shown in the MMG activity.	Randomised, controlled trial.	Unable to identify the location where the neural plasticity process took place.
[91] ES45	Cattagni et al. 2018	To investigate the acute effect of unilateral NMES on the knee extensors of the right leg on isometric MVC, surface EMG (VL&RF-agonist and BF-antagonist) and voluntary activation (twitch interpolation) of the left leg; and to examine the potential dose-response relations between the NMES intensity (None, Low = 10%MVC and High = 30%MVC) and contralateral strength gain in healthy young men. The ES was delivered with the pulse width of 400 μs, frequency of 50 Hz and intensities that induced none, 10% or 30%MVC for 5 s, with 3 contractions at each intensity.	The MVC, voluntary activation and VL and RF EMG were higher for High-intensity, and VL EMG was higher for both Low- and High-intensity NMES, and RF EMG for High-intensity was higher than the None condition.	MVC and indicators of voluntary activation were examined at the same time, and EMG of the antagonist was also examined.	The evoked %MVC was not re-checked during testing and compared to the responses to voluntary contraction.
			There was no difference between the Low and High NMES conditions, i.e. no dose-response relationship was observed.		EMG was only recorded from VL and RF muscles.
					There was a lack of an active control condition.
[97] ES51	Benito-Martínez et al. 2020	To determine whether unilateral application of NMES could result in local and cross-education thermal effects, and the duration of the effects, in healthy young adults. Participants received NMES with the pulse width of 400 μs, frequency of 8 Hz, and intensity of maximum tolerance for 12 min.	A temperature cross-education effect was produced, and the effect was greater when the stimulation was applied on the dominant side. The cross-education effect in the contralateral leg lasted for up to 10 min post stimulation.	A single group of participants with the NMES applied to either dominant or non-dominant side in a random order (1:1).	Only applied a stabilisation period of 10 min prior to NMES.
					No control for the potential effects of food intake and menstrual cycle in female participants.
[100] VB1	Jackson et al. 2003	To investigate the acute effect of vibration on the right rectus femoris muscle on isometric knee extension MVC of both legs in healthy young men. Participants received vibration with the amplitude of 1.5–2.0 mm and frequency of 30 Hz and 120 Hz (on different days) for 30 min.	The unilateral vibration at 30 Hz and 120 Hz both resulted in a significant reduction of MVC and rate of force generation in both limbs, whilst no significant changes in EMG of the rectus femoris (except in right leg) and vastus lateralis of both legs.	A single group of participants, with muscle strength and surface EMG measured pre and post the intervention.	Not stated.
[105] VB7	Karacan et al. 2012	To investigate whether bone mineral density or bone mineral content of the ultradistal radius has an effect on the resting muscle activity of contralateral wrist flexor muscles during unilateral forearm vibration in healthy adults. Vibration was applied to the right (dominant) arm with the vibration load of 1/3 of the ideal body weight in women (+3 kg in men) and frequency of 46 Hz for 1 min.	The EMG of the left wrist flexor muscles significantly increased during vibration of the right arm. Multiple linear regression analysis revealed that the right ultradistal radius bone mineral density was an independent predictor of the resting EMG activity of the left wrist flexor muscles measured during vibration.	A single group, self-controlled, double-blind trial to examine the potential relationship between the bone mineral density and content and EMG responses to unilateral vibration.	The total number of osteocytes per unit volume was not calculated. Young’s modulus of the cases was not calculated.
				No muscular strength was assessed in relation to the cross-education effect.	
[108] VB10	Marín et al. 2014	To investigate the acute effects of unilateral whole-body vibration on the dominant leg on the performance of explosive leg press at 40%MVC, and EMG of the vastus lateralis and medial gastrocnemius of the contralateral leg in healthy young men. Participants received vibration at a high amplitude at 50 Hz, a low amplitude at 30 Hz, or no vibration (sham), for 30 s.	The vibration at 50 Hz resulted in a greater increase in the mean velocity of the stimulated leg at 2-min post, and that of the unstimulated leg immediately post and at 2-min post, compared to 30 Hz and sham.	A single group of participants was treated with three conditions separately in a random order.	No MVC and other neuromuscular performance variables were assessed post vibration.
			There were no changes in the EMG of both legs.		Only used two vibration stimuli.
					Only investigated healthy young male participants.
[110] VB12	García-Gutiérrez et al. 2018	To investigate the acute effects of form roller massage with and without vibration, and no form roller massage (control), on plantar flexors of the dominant leg, on the isometric MVC of the dorsiflexion and plantarflexion, and ankle dorsiflexion mobility in healthy young adults. Participants received vibration with the amplitude of 1.95 mm and frequency of 49 Hz for 20 s.	No significant changes were found in plantar flexion and dorsiflexion strength in response to the treatment, while the ankle dorsiflexion range of motion was higher in both treated groups than that in the control, in both the treated and the contralateral legs.	A single group, self-controlled trial, with the three conditions performed in a randomised order.	Not stated
[23] VB13	Minetto et al. 2018	To investigate the acute effects of NMES, and focal vibration on the right quadriceps, on isometric knee extension MVC of the left leg in healthy men. Participants received NMES with the pulse width of 400 μs, frequency of 50 Hz and intensity that induced 30%MVC for 10 s; and vibration with the pressure of 336 mbar and frequency of 300 Hz for 5 min; or no stimulation or vibration.	The MVC and voluntary activation of the left quadriceps increased during contralateral NMES and vibration, with remarkable inter-individual variability (responders).	A single group, self-controlled trial.	Not stated
		Voluntary activation (twitch interpolation) and EMG were measured.	The increases in voluntary activation and EMG elicited by NMES were higher than those elicited by focal vibration.		
[114] VB18	Delkhoush et al. 2020	To evaluate the acute effects of unilateral whole-body vibration on EMG of four forearm muscles and grip strength of the contralateral hand in healthy young adults. Participants received vibration with the amplitude of 2.5 mm and frequency of 35 Hz for 3 min.	No significant change was observed in either the EMG of the forearm muscles, or the grip strength of the contralateral limb.	A single group of participants with a randomised crossover design.	Only measured EMG from four muscles in the forearm, and the grip strength changes of the contralateral limb.
					Only applied one session of vibration at 35 Hz.
**Chronic effect**					
[52] ES3	Cabric et al. 1987	To determine the cross-transfer effects of 3 weeks of unilateral electrical stimulation training on maximal isometric plantar flexion force in healthy young men. Participants in the training groups received ES with the pulse width of 200 μs, frequencies of 50 Hz for group I, and 200 Hz for group II, and incremental intensity of 40 to 45 mA, decremental duration of 50 s to 20 s, 15 to 25 contractions per day, for 21 days.	Both stimulation programs resulted in a significant increase of contraction force in both limbs.	Randomised, controlled trial, with measurements of skinfold and calf girth.	Not stated
			Calf girth was increased significantly in the stimulated limb but not in the contralateral limb. Dorsal calf skinfold decreased significantly in the stimulated leg but not in the non-stimulated leg.		
			The control group showed no change in any of the measurements.		
[54] ES5	Lai 1988	To investigated the effects of 3 weeks of EMS training of the left quadriceps femoris limb on the strength of the unstimulated right limb in healthy young men and women. Participants of the training groups received EMS with the pulse width of 200 μs, frequency of 50 Hz, and intensities that induced 50% isometric MVC (HI) or 25%MVC (LI), 5 s on 5 s off, for 3 sets of 10 contractions in each session, 5 sessions per week for 3 weeks.	The isometric knee extension strength significantly increased in response to both training intensities and in both limbs, with the HI group showed significantly greater strength gain than the LI group in the trained limb; while no significant difference found in the contralateral limb between the two groups.	Randomised, controlled trial, with measurements of both isometric and isokinetic strength. Carry-over effects were evaluated at three weeks post training.	Not stated
			The isometric MVC of the HI group measured at the end of the three-week follow-up period was still higher than that of pre training in both limbs.	Equal number of male and female participants.	
			The isokinetic concentric strength (60 deg/s) was also measured, with a significant increase in the stimulated limb in both groups post training, while no significant change was found in the contralateral limb.		
			No significant changes were found in the control group.		
[57] ES8	Tachino et al. 1989	To investigate the effects of 6 weeks of unilateral EMS on the tibialis anterior when the muscle is maximally stretched or shortened, on the strength of the contralateral ankle dorsiflexors in healthy women. Participants received EMS with the pulse width of 200 μs, frequency of 50 Hz, and the intensity of maximum tolerance, 10 sets of 10 s stimulation per day, 4 sessions per week, for 6 weeks.	The isokinetic torque of ankle dorsiflexion increased significantly in the stimulated limb of both the shortened and stretched groups after training, while the stretched group showed greater strength gain.	Compared cross-education effects when the muscle was stretched or shortened. The sample was not randomly assigned to the two groups. There was no blank control group.	Not stated
			However, in the contralateral limb, only the stretched group showed a significant strength gain after 2 weeks of training.		
[62] ES13	Hortobágyi et al. 1999	To compare the contralateral (untrained right leg) and ipsilateral (trained left leg) adaptations in knee extension muscle strength under voluntary and stimulated conditions, pre and post 6 weeks of eccentric training in young women. Participants were randomly assigned to a voluntary, an EMS, a remote EMS (on left arm, during voluntary leg contractions), and a control group. Isometric and eccentric knee extension strength of both legs under both voluntary and stimulated conditions were assessed. Hand grip strength was also assessed to examine whether the cross-education occurred in homologous muscle only. The EMS group trained with stimulation frequency of 2,500 Hz, 50 bursts/s, 50% duty cycle and intensity to maximum tolerance, incrementally 4–6 bouts of 6–8 reps per session, 4 sessions per week for 6 weeks.	The strength gain of EMS-evoked contraction was greater than that in voluntary contraction in all training groups.	Randomised, controlled trial.	Not stated
			The EMS and rEMS training caused greater cross-education than voluntary training.	Participants were females.	
			Strength gain tested under eccentric mode was greater than that under isometric mode.	A remote EMS group was included to examine the potential mechanisms of cross-education.	
			Contralateral strength gain was the greatest in the eccentric test in the EMS group.	Both voluntary and stimulation evoked contractions were assessed.	
			Both isometric and eccentric strength tests were used to examine the potential specificity of the training and testing.	EMG of both legs increased after training.	
			No significant change found in grip strength.	Surface EMG was recorded from VL and VM.	
[64] ES15	Zhou et al. 2002	To investigate the effects of 4 weeks unilateral EMS and voluntary training on the dominant leg on the knee extension strength of both legs in healthy men. The EMS group trained the dominant leg with pulse width 250 μs, frequency of 100 Hz, and intensity that induced 65%MVC, for 40 isometric contractions with 5 s on 20 s off cycles in each session, 3 sessions per week for 4 weeks.	The isometric knee extension strength significantly increased in both limbs of both the EMS and voluntary training groups, while the isokinetic torque (60 deg/s, 180 deg/s) only showed significant improvement in the trained limb but not in the untrained contralateral limb.	A sample of convenience was assigned to an EMS, a voluntary training and a control groups.	Not stated
			Surface EMG did not show a significant increase in either limb.	Isometric and isokinetic strength were tested for the specificity of the training effect.	
			No significant changes were found in the control group.		
[71] ES22	Yu et al. 2008	To investigate the bilateral effect of 6 weeks unilateral EMS and voluntary isometric training on ankle dorsiflexion strength and muscle activation (twitch interpolation) in healthy young men. The EMS group trained with pulse width of 200 μs, frequency of 50 Hz, and intensity that induced 60–70%MVC, 5 s on 10 s off cycles for incremental 3–5 sets of 8 contractions per session, 3 session per week for 6 weeks.	The isometric dorsiflexion strength significantly increased after training in both limbs of both the EMS and voluntary training groups, and the muscle activation was significantly improved in both limbs of the EMG group but not in the voluntary training and control groups.	Randomised, controlled trial, with muscle strength and activation measured pre and post training.	Not stated
[12] ES23	Bezerra et al. 2009	To investigate the bilateral effects of 6 weeks unilateral training on the right leg with EMS superimposed on maximal voluntary contraction (EVG), and maximal voluntary contraction only (VG), on isometric knee extension strength in healthy men. The EVG group trained with pulse width of 400 μs, frequency of 100 Hz, and intensity of maximum tolerance, 5 s (plus 1 s ramp-up and 1 s ramp-down) 5 off, for 3 sets of 10 contractions, 3 sessions per week for 6 weeks.	The EVG group demonstrated significant increase of isometric strength in both limbs, while that of the VG only increased in the trained limb.	Randomised, controlled trial.	Not stated
			The quadriceps cross sectional area increased significantly in the trained limb of both EVG and VG, while no significant change was found in the contralateral limb.	Assessment of EMG and muscle cross sectional area using MRI.	
			The control group showed no significant change.		
[77] ES29	Sariyildiz et al. 2011	To evaluate the effect of 6 weeks training with EMS induced eccentric contraction of the dominant wrist flexors on the isokinetic torques of both arms, including muscle strength of the contralateral wrist extensors in healthy men. The EMS group trained with pulse width of 250 μs, frequency of 85 Hz for 4 s with 1.5 s rise time and 0.75 s fall time, and intensity of maximum tolerance, for 20 min, 5 sessions per week for 6 weeks. The control group received TENS with pulse width of 50 μs, frequency of 100 Hz and intensity that the participants felt comfortable paraesthesia with no muscle contraction for 20 min in each session.	Similar strength gains were found for both wrist flexor and extensor muscles in both arms of the EMS group.	Randomised, controlled trial with measurement of strength from both the wrist flexors and extensors.	Small sample size, 12 in EMG group and 11 in the control group.
			No significant changes were found in the TENS group.		
[80] ES32	Popa et al. 2012	To investigate the effect of 10 days unilateral FES on motor symptoms in Parkinson’s patients compared to healthy controls. FES was applied to the radial nerve and common peroneal nerve of the more affected side, with pulse width of 300 or 350 μs (used different devices), frequency of 40 Hz, and intensity of 10–100 mA, 30 min per day for 10 days.	The intervention improved motor functional test (e.g., Schwab & England scale).	Unilateral FES was applied to both an arm and a leg.	Not stated
			The cross-education effect seemed to be more pronounced in the Parkinson’s patients than that in the healthy controls.		
[20] ES36	Onigbinde et al. 2014	To investigate the cross-education effect of 8 weeks unilateral TENS in healthy young men and women. Stimulation was applied on the right quadriceps femoris, with pulse width of 100 μs, frequency of 85 Hz and intensity of maximum tolerance, 15 min per session, 2 sessions per week for 8 weeks.	The isometric strength of the quadriceps in post training test was significantly greater than that in pre training test in both limbs.	A sample of convenience, single group, self-controlled trial.	The sample size was relatively small (50).
			There was a significant increase in the girth of the stimulated limb.		Limb girth was measured for the stimulated side only.
[19] ES44	Kadri et al. 2017	To compare cross-education effects of 8 weeks NMES and voluntary isometric knee extension training on muscle strength and monopedal postural control in healthy young men. The NMES group trained the quadriceps of the non-dominant limb with pulse width of 380 μs, frequency of 50 Hz and intensity that induced 20%MVC, 7 s on 7 s off for 10 min in each session, 3 sessions per week for 8 weeks.	The isometric MVC was improved similarly for both the voluntary and NMES training groups and in both limbs, while the postural control showed no significant improvement.	Randomised controlled trial.	Not stated
[96] ES50	Barss et al. 2020	To determine the relative contribution of cutaneous afferent pathways as a mechanism of cross-education by directly assessing if unilateral cutaneous stimulation alters ipsilateral and contralateral strength gains in wrist extensors in healthy young adults. Participants were randomly assigned to voluntary training (TRAIN), cutaneous stimulation (STIM, twice of radiating threshold, 3 s, 50 Hz) to the superficial radial nerve, and TRAIN+STIM groups, 6 sets of 8 reps, 3 sessions per week for 5 weeks.	TRAIN and the TRAIN+STIM groups showed significantly higher wrist extension torque gain than that of the STIM in the trained limb. The TRAIN group also showed significantly higher torque of the untrained limb compared with the other two groups post training.	Randomised group allocation.	The timing and the intensity of the electrical stimulation applied might not be “natural”.
			There were no significant changes in muscle activity (EMG), wrist flexion torque, and handgrip strength post training in both limbs in all groups.	To determine the effect of cutaneous stimulation at the intensity of 2 x radiating threshold on cross-education.	Unable to assess the effect of cutaneous stimulation to the superficial radial nerve during wrist extension contractions had on the peak force production within each training session.
			Voluntary wrist extension training or repeated electrical stimulation to a cutaneous nerve does not appear to alter cutaneous reflex transmission across contraction intensity or latencies of response.		
			However, receiving a large sensory volley during wrist extension training altered long-latency cutaneous reflex amplitude from inhibition to facilitation at high levels of muscle contraction on the trained right side.		
[99] ES53	Yurdakul et al. 2020	To evaluate the effects of adding EMS to wrist flexor muscles on the nonparetic limb in conventional stroke training to strengthen homologous agonist and antagonist muscles on the paretic side in patients with subacute stroke. All patients underwent 40 min lower limb training, and 20 min stretching exercise for the paretic upper limb. The patients in the EMS group received 30 min electrical stimulation to their nonparetic forearm wrist flexors, with pulse width of 250 μs, frequency of 85 Hz and intensity to patients’ tolerance for 6 s on 10 s relaxation (with 4 Hz frequency) cycles; and those in the TENS group received 30 min stimulation with pulse width of 50 μs, frequency of 100 Hz at intensity of the sensible threshold; 5 sessions per week for 6 weeks.	The EMS and TENS groups improved similarly in the functional tests.	A clinical trial that applied EMS on the non-paretic limb to investigate the benefits of cross-education in subacute stroke patients.	The sample size was small (15 in each group).
			The EMS groups showed a greater increase in the wrist flexion force than the TENS group, while no significant difference was found in the wrist extension force of the paretic limb.		Only included relatively recovered upper extremities of patients with sub-acute stroke.
[103] VB4	Couto et al. 2012	To investigate the cross-education effects of 4 weeks unilateral isometric MVC training with and without mechanical vibration on isometric strength of elbow flexors in healthy young men. All participants performed elbow flexion for 6 s MVC, with 12 repetitions in each session (the limb trained and the number of sessions each week were not reported) for 4 weeks. The Vibration group received local vibration with amplitude of 6 mm and frequency of 8 Hz during the MVC.	The isometric elbow flexion strength increased in both the trained and untrained arms of both groups, while the Vibration group showed significantly higher strength gain than that of the MVC only group.	Randomised group allocation.	Not stated
			EMG of the trained biceps in the Vibration group was significantly higher than that of the MVC only group.	The strength of the elbow flexors and EMG of both elbow flexors and extensors were measured.	
			No significant differences were found in other elbow flexor or extensor muscles.		
[21] VB6	Goodwill et al. 2012	To investigate the cross-education effects in response to 3 weeks of unilateral squat training on the right (dominant) leg with and without superimposed whole body vibration, in healthy young adults. The strength training (ST) and ST plus whole-body vibration (ST+V) groups performed single leg squats with 3 sessions of 4 sets of 8 reps with 75%, 77.5% and 80% 1RM load in week 1, 2 and 3 respectively. The ST+V group received vibration during training with displacement of 2.5 mm and frequency of 35 Hz.	The dynamic single leg 1RM strength increased significantly after training in both legs compared to the control group, with no significant difference found between the ST and ST+V groups. No difference was found between the two training groups in the peak height of recruitment curves or short-interval intracortical inhibition (by TMS).	Randomised, controlled trial, with measurement of muscle strength, and corticomotor plasticity of the ipsilateral side.	Future studies may consider applying individualised gravitation load; and measures of corticomotor adaptations contralateral to the trained limb.
			There was a main effect of (training) time for muscle thickness of the trained leg, but not for the untrained leg, neither for group by time interactions.	Muscle thickness was measured by ultrasound.	The effect on spinal reflexes was not investigated.
[107] VB9	Lapole et al. 2013	To investigate the effect of 14 days of unilateral Achilles tendon vibration on isometric plantar-flexion MVC, H-reflex and V-waves of the soleus and gastrocnemius of both legs in healthy young adults. Vibration was applied on the right limb with amplitude of 1 mm and frequency of 50 Hz, 1 hr daily for 14 days.	The MVC increased in both legs after the 14 days vibration. The H-reflex increased in the vibrated soleus but not in the contralateral side. The V-wave increased on both sides. The V-wave also increased in the gastrocnemius medialis of both legs but not in the gastrocnemius lateralis.	Single group, self-controlled.	Not stated
				Muscle strength and H-reflex were measured.	
[109] VB11	Souron et al. 2017	To investigate the effect of 8 weeks local vibration training on the right tibialis anterior, on isometric dorsiflexion MVC of both legs, after 4 and 8 weeks of training, and at 2 weeks post the training, in healthy young adults. Cortical voluntary activation was evaluated by TMS (evoked superimposed twitches). The vibration group received local vibration with amplitude of 1 mm and frequency of 100 Hz, 1 hour per session, 3 sessions per week.	The vibration training significantly increased MVC in both legs after 4 and 8 weeks of training and at 2 weeks post training. The cortical activation was significantly increased for both legs, whilst no changes were found in MEP and cortical silent period.	Randomised, controlled trial, with MVC and central voluntary activation assessed during and post the training.	MEP recorded at 50% and 75% MVC may not reflect the corticospinal excitability during maximal contraction.
[113] VB17	Aydin et al. 2020	To determine whether 4 weeks unilateral whole-body vibration training induced strength gain in the untrained contralateral leg muscle and the potential role of spinal neurological mechanisms in healthy men. The vibration group placed the right leg on the platform in static semi-squat position and received incremental vibration at amplitude of 1.1–2.2 mm and frequencies from 30 to 45 Hz for 30 to 60 s at each frequency for 4 to 8 min, 5 sessions per week for 4 weeks. The Sham group received vibration with reduced acceleration (reduction of 99.52 to 99.93%).	There was a significant increase of in knee extension strength after training in both the vibrated and non-vibrated limbs in the vibration exercise group, while no significant changes were found in the Sham control group. The vibrated leg showed a shorter vibration-induced muscle reflex latency than that of the non-vibrated leg.	Randomised, sham-controlled, triple-blind design.	The isokinetic strength test was performed on the right (vibrated) leg first, which might have an effect on the subsequent test on the left (non-vibrated) leg.
[41] ND3	Huang et al. 2007	To investigate the effect of 4 weeks unilateral electroacupuncture at two acupoints on the tibialis anterior muscle on isometric ankle dorsiflexion MVC of both legs in healthy young men. Needling was applied to the ST-36 and ST-39 acupoints of the right leg, with pulse width of 1 ms, frequency of 40 Hz and intensity of 30–40 V, for 8 duty cycle of 1 min on 1 min off, 3 sessions per week for 4 weeks.	The dorsiflexion MVC of both legs significantly increased after the four weeks of electroacupuncture, while the control group showed no change.	Randomised, controlled trial.	Not stated
[24] ND4	Zhou et al. 2012	To compare the effects of 6 weeks unilateral training with manual acupuncture (MAcu) and electroacupuncture (EAcu) on two acupoints, ST-36 and ST-39, and sham points (ESham) in the tibialis anterior of the right leg on isometric ankle dorsiflexion strength of both limbs in healthy young men. All participants of the treatment groups received needling incrementally from 15 to 30 min, 3 sessions per week for 6 weeks. The MAcu group received needle twirling and lift-thrusting, and the electroacupuncture groups received electrical stimulation via the needles with pulse width of 1 ms, frequency of 40 Hz and intensity of maximum tolerance. The control group performed the same warm-up and cool-down activities as the treatment groups, but otherwise rested in the lab for the same time period.	The dorsiflexion MVC increased significantly in both limbs after the needling treatment in all groups except the control group.	Randomised, controlled trial.	A group of unilateral manual acupuncture on sham points was not included.
					The participants were healthy young men. Further studies are needed to confirm the therapeutic effect in patients or ergogenic effect in resistance-trained individuals such as athletes.
[25] ND7	Huang et al. 2015	To investigate the effect of 8 weeks unilateral manual acupuncture (MAcu) and electroacupuncture (EAcu) on two acupoints, ST-36 and ST-39, or two non-acupoints (MSham and ESham) in the tibialis anterior muscle of the right leg, on isometric ankle dorsiflexion MVC, and muscle activation (twitch interpolation), of both legs in healthy young men. The manual needling groups received twirling and lift-thrusting and the electroacupuncture group received electrical stimulation via the needle with pulse width of 1 ms, frequency of 40 Hz and intensity to the maximum tolerance, incrementally 15–30 min per session, 3 sessions per week for 8 weeks. The control group performed the same warm-up and cool-down activities as the treatment groups, but otherwise rested in the lab for the same time period.	Needling on acupoints or non-acupoints, with or without electrical stimulation, resulted in similar strength gains, as well as in muscle activation, in both the stimulated and non-stimulated legs, after eight weeks of intervention, and the strength gain sustained for at least three weeks after the intervention.	Randomised, controlled trial with comparisons between treatments on acupoints and non-acupoints, and with and without electrical stimulation, together with assessment of muscle activation.	Not stated
		Follow-up tests were performed at 2 and 3 weeks post intervention.		Monitored the carry-over effect for 3 weeks.	

Keys: CSP = cortical silent period; EMG = electromyography; EMS = electromyostimulation; ES = electrical stimulation; MEP = motor evoked potential; MVC = maximal voluntary contraction; MRI = magnetic resonance imaging; MVC = maximal voluntary contraction; NMES = neuromuscular electrical stimulation; TENS = transcutaneous electrical nerve stimulation; TMS = transcranial magnetic stimulation.

## Discussion

There were five major findings from this scoping review.

### Research that addresses the effect of unilateral peripheral stimulation should consider the potential cross-education

Although there appears to be a broad research interest in investigating the effects of unilateral peripheral stimulation, there were only 31 out of the 83 studies reviewed (37.3%) claiming that their studies were primarily within the context of cross-education (Table 4). Other studies investigated the effects of unilateral stimulation while using the contralateral side as a control (13, 15.7%), examined the impact of unilateral stimulation on cortex activities or plasticity (24, 28.9%), or examined the clinical efficacy of unilateral interventions (15, 18.1%) (Table 2). This indicates that many researchers may not be aware of the phenomenon of cross-education induced by peripheral stimulation, while investigating the effects of unilateral interventions. Therefore, the cross-education phenomenon should be introduced to the broader research community. Particularly, investigators who would use the contralateral side as a within-subject control for unilateral interventions should consider the potential cross-education effect in future studies.

It should also be noted that few studies aimed to investigate the cross-education effect induced by peripheral stimulation on motor skills. This might be due to the involuntary nature of the peripheral neuromuscular stimulation, or the search terms used in this review that did not specifically focus on motor skill aspects, that is a limitation of this scoping review. A pertinent question of interest is whether unilateral peripheral neuromuscular stimulation should be hypothesised as affecting the contralateral side’s skill performance. This also underscores the recommendation from the recent Delphi survey that the context of a study, e.g. transfer of strength or skill, should be clearly presented in future reports [3].

### Chronic unilateral peripheral neuromuscular stimulation appears to cause robust cross-education effect on motor performance

A thorough analysis of the outcomes of the above-mentioned 31 studies on cross-education is beyond the scope of this review and may be addressed in a separate systematic review or meta-analysis. Briefly, 21 studies investigated chronic effects of unilateral peripheral stimulation (Table 4), of which all demonstrated a significant increase in motor performance of the contralateral limb. In contrast, among the 10 investigations on the acute effects, two studies reported no significant changes in strength or EMG in the unstimulated contralateral side [109, 114], whist another study [100] reported a reduction in MVC in both limbs but no change in EMG after a short period of vibration. Therefore, the chronic effects of unilateral peripheral stimulation on motor performance of the contralateral side appear to be robust, while the acute effects might be inconsistent (particularly in response to vibration) possibly due to the differences in research design and outcome measures (Table 4).

The majority of the 83 studies investigated healthy participants (58, 69.9%). In contrast, the studies on patients were limited, with 12 (14.5%) on CNS disorders such as post-stroke recovery, and 13 (15.7%) on patients post surgical operation or injuries, with one studying both patients and healthy individuals. Only 16 studies (15.7%) were conducted in clinical settings, and only two of these studies investigated the cross-education effect in patients. This finding is similar to that from a recent meta-analysis that identified only six studies on patient populations out of 96 studies reviewed (including unilateral voluntary or ES interventions) [6], indicating a lack of clinical studies on the application and efficacy of cross-education interventions. Therefore, studies on the translation of the cross-education effects found in healthy populations to clinical practice should be enhanced.

### The physiological mechanisms underlying the cross-education induced by peripheral neuromuscular stimulation are unclear

It has been speculated that the sensory inputs would play a major role in manifesting the contralateral changes in response to peripheral neuromuscular stimulation [1, 36]. The cross-education caused by peripheral neuromuscular stimulation possibly cannot be explained by the hypothesised mechanisms for unilateral voluntary resistance training [15], and the exact pathway/s and mechanism/s remain to be elucidated.

There have been numerous studies that investigated the CNS responses or plasticity to unilateral peripheral stimulation, utilising a variety of methods and techniques, including EEG (2), EMG (35), fMRI (7), fNIR (2), MEG (1), reflexes (11), TMS-MEP (9), and central activation (twitch interpolation, 5). Bilateral cortical activation and/or changes in neural plasticity and muscle activation were reported in several studies but they only investigated acute effects, e.g. [65, 73, 76]. Few studies have attempted to identify what and how sensory inputs are involved in the manifestation of the contralateral effect [96], possibly due to the lack of a suitable methodology.

It is hypothesised that the mechanism of the cross-education, induced by the peripheral neuromuscular stimulation, is based on adaptations in the CNS [12, 21] because there is no significant change in muscle size of the unstimulated contralateral side. This review found nine studies that measured muscle morphological changes, such as limb girth (1), muscle cross sectional area or thickness (1 CT, 1 MRI, 4 ultrasound), or muscle fibre cross-sectional area and fibre type composition (2 histochemistry) (Table 3). However, among the studies that claimed to examine the cross-education effect, there was only one study that measured changes in muscle cross-sectional area using MRI in response to a training program with EMS superimposed on voluntary exercise [12], and one study assessed CNS activity together with muscle thickness measured by ultrasound in response to a vibration training program [21]. Therefore, there has been no sufficient evidence on whether the unilateral peripheral neuromuscular stimulation would affect muscle size in the unstimulated contralateral side comparing with the stimulated side, although it may be predictable that muscle hypertrophy would not occur in the unstimulated limb in response to a short period of intervention.

### Unilateral intramuscular needling can affect muscle strength of the contralateral limb

The concept of contralateral treatment for ipsilateral health conditions using needling has historical roots. More recently, an increased number of investigations utilising randomised and controlled trial designs to examine its clinical efficacy and or mechanisms have emerged [30, 31]. However, much of the research in this area is not published in English. This scoping review has identified 12 studies on the effect of unilateral needling with three specifically addressing the cross-education effect on muscle function [24, 25, 41]. The Tianjin University of Sport and Southern Cross University research group has applied unilateral needling to the tibialis anterior muscle in three trials on healthy young men, with intervention durations from 4 to 8 weeks. These trials reported robust and similar bilateral strength gains, regardless of whether the needles were applied on known acupoints or sham points, or whether the needles were manually operated or electrical stimulation was delivered through them [24, 25, 41]. Other laboratories are encouraged to undertake similar investigations to confirm the phenomenon and conduct trials on clinical populations to evaluate the clinical efficacy of unilateral needling. There have been systematic reviews and meta-analyses on the effects of unilateral needling therapy for various patient groups, such as post stroke or injury [30, 31]. However, due to the language barrier, most of these studies are not indexed by the English-based databases. The physiological mechanisms for the effects of needling remain unclear [32, 33], particularly for the needling without electrical stimulation. It has been reported that unilateral manual needling on limb muscles resulted in modulatory effects in human brain as shown in functional MRI [122], and electroacupuncture caused bilateral changes in the insulin-like growth factor (IGF-1) mRNA and protein in rat brain of an ischemic stroke model [123]. However, it has also been reported in a study on rats that unilateral ES significantly increased the level of IGF-mRNA in the stimulated muscle but not in the unstimulated muscle of the contralateral side [124]. Obviously, further research is required to explore the neural or other mechanisms underlying the effects of needling interventions on motor performance. This area of investigation would benefit from cross institutional collaboration to replicate and translate (to clinical practice) past findings, examine clinical efficacy, and elucidate potential mechanisms of cross-education caused by needling.

### Demographic characteristics of the research

#### Research design

Although it is beyond the scope of a scoping review to evaluate the quality of the included studies [37], a few important aspects should be briefly discussed. Various designs were employed in the 83 studies, with 30 utilising a randomised, controlled design and 40 utilising a single group for pre vs post intervention comparison. The use of a single group for within-subject comparison may be very well justifiable according to the study’s objective. However, more randomised, controlled clinical trials on patients or specific populations would be required to confirm the clinical efficacy or implications of unilateral/contralateral intervention/therapy in the context of cross-education.

It was also noted that 82 out of 83 studies employed P-value-based statistics (one other study was a single case report), with 12 studies reported the effect size (although not necessarily for magnitude-based assessment). However, only 13 studies presented a statistical justification for the sample size, and 30 studies reported justifications for the use of the statistical analysis against the method’s assumptions (e.g. ANOVA). These statistical issues should be carefully considered when designing future studies to minimise ambiguous findings. It is noted that the majority of the studies that reported the justification of sample size and against the assumptions of the method was published from 2010 onwards (Table 3), reflecting the trend of increased rigor in research reports.

#### Location of the study

Of the 83 studies reviewed, 50 (60.2% were published within the past 10 years, indicating an increased research interest in this area (Table 2). Studies were undertaken by researchers/research groups from all continents (except Antarctica), with 13 studies coming from the United States (15.7%), 11 from China (mainland, 13.3%), 6 (7.2%) from each of France and Spain, and 5 (6.0%) from each of Australia, Brazil, Canada and Turkey (Table 2). The remaining articles were from other 15 countries or regions, with each having one to four publications. Although these counts were based only on the first author’s first affiliation, it indicates a wide spread interest from researchers worldwide.

#### The participants

The studies included in this review recruited participants from a wide age range (14–89 years), with approximately 70% of participants being males. A majority of the investigations recruited young adult participants (approximately 50 studies focused on participants under 35 years of age); while a relatively small number of studies investigated on specific populations, e.g. 24 studies involved participants over 60 years of age, and two recruited female participants only (Table 2). Thirteen (13) studies did not specify gender profile, and one study did not report participants’ age (Table 2). The reporting of demographic information should be considered to be essential in future studies for an accurate and clearer description of the participants or the population concerned. It is known that the neuromuscular function changes with ageing (e.g., muscle strength, fatiguability, mass and fibre type composition), as well as in response to various health conditions, diseases and interventions [125–128]. Whether there is an age- or gender-related difference in peripheral neuromuscular stimulation induced cross-education cannot be determined from the current literature due to the limited evidence available. Further studies in these aspects would advance our understanding on cross-education and may inform clinical implications as well.

#### The ipsi- and contra-lateral side investigated

Previously there have been debates on whether a cross-education effect is influenced by limb dominancy or asymmetry in response to voluntary interventions [5, 129–131]. Such debates have not principally focused on the effects of unilateral peripheral stimulation. However, it is of note to find in this scoping review that more studies had stimulated the dominant side (14) compared to the non-dominant side (2), had investigated the affected or weaker side (21, mainly for patients) than the less affected side (2), or had utilised the right side (28, that may or may not be the dominant side) than the left side (6); there were three studies that stimulated the right and left side alternately, and four studies did not indicate how/why one side was chosen to be stimulated (Table 3). Whether the cross-education effects induced by peripheral neuromuscular stimulation are influenced by limb dominancy or asymmetry would be an interesting topic for future studies.

Furthermore, considering the potential clinical implications of the contralateral effect caused by unilateral intervention as repeatedly suggested in the literature, it would be necessary to evaluate the efficacy of unilateral interventions on the less affected limb in the treatment or rehabilitation of the more affected side. However, most (21/25) of the studies on patients included in this review applied the stimulation on the more affected limb, whilst only one study that applied chronic ES on the nonparetic limb in patients with subacute stroke [99] to investigate cross-education effect. Therefore, further studies are needed to determine the efficacy of unilateral interventions on the less affected side, with cross-education being used as the theoretical framework in specific and clinical populations.

#### The subject muscles

In respect to muscle groups examined, 104 muscles or limbs were tested in the studies reviewed. Among them, 61 (58.7%) stimulated muscles were in leg with 30 on knee extensors, 16 on ankle dorsiflexors, and 10 on plantar flexors; 40 (38.5%) stimulated muscles in arm; and other studies stimulated the paraspinal, neck or hip muscles (4), or on one or more points on a limb (10, such as needling or vibration) (Table 3). Over one-half of the 83 studies (48, 57.8%) assessed the effect on the homologous muscles contralateral to the stimulated side. In contrast, most of the remaining studies (31, 37.3%) examined the CNS responses (Table 3), and a small number of studies also assessed the responses in clinical functional assessments [68, 93, 99], spasticity [59], sympathetic nervous system function [61] or cutaneous thermal regulation [97]. Among the 31 studies that claimed to investigate cross-education, 15 were on knee extensors, six on ankle dorsiflexors, six on forearm muscles, three on plantar flexors, and one on elbow flexors. The accessibility of the muscles might be a major factor for such a distribution. However, whether the homologous muscles in the arms and legs, and/or muscles with different functionality (e.g. muscles in hands vs those in legs, with different motor unit sizes or types) would respond to unilateral peripheral stimulation differently are unknown and require further investigation.

It is interesting to note that some studies have examined the effect of unilateral stimulation on the function of the autonomic nervous system, for example, thermal regulation [97], blood pressure and heart rate [61], or pain [74, 86]. However, these measurements do not fit in the definition of cross-education (on motor outputs or skill).

## Summary and recommendations for future studies

An increased research interest in cross-education is seen over the past decades in the area of contralateral effects of unilateral peripheral neuromuscular stimulation, such as neuromuscular electrical stimulation, focal or whole-body vibration, and needling. However, only one-third of the studies were designed to examine cross-education specifically, and many studies utilised the contralateral (unstimulated) limb as a within-subject control. Considering the strong evidence for the cross-education phenomenon, a potential methodological flaw may exist when using a within-subject (contralateral) control design. Therefore, it is important that the broader neuromuscular physiology research community be made aware of the contralateral effect of unilateral stimulation.

Few studies on patients included in this review applied stimulation on the less affected side to investigate the clinical implications or applications of cross-education. Future research with randomised, controlled clinical trials on patients and/or specific populations is required to determine the clinical efficacy for applying unilateral peripheral stimulation as a means of intervention.

The majority of the studies employed electrical stimulation, while other types of stimulation (such as vibration and needling) are emerging and demonstrate detectable cross-education effects. In respect of the underlying mechanisms, it has been speculated that peripheral sensory inputs play a major role in the manifestation of the contralateral effect. However, the current literature has not clearly identified, or hypothesised, which sensory pathway/s is/are most relevant and effective to cause central plasticity and/or the contralateral effect on motor performance in response to acute and chronic peripheral neuromuscular stimulation.

## Supporting information

S1 Checklist PRISMA-ScR-checklist. (DOCX)
